# Niosomes for Topical Application of Antioxidant Molecules: Design and In Vitro Behavior

**DOI:** 10.3390/gels9020107

**Published:** 2023-01-26

**Authors:** Maddalena Sguizzato, Alessia Pepe, Anna Baldisserotto, Riccardo Barbari, Leda Montesi, Markus Drechsler, Paolo Mariani, Rita Cortesi

**Affiliations:** 1 Department of Chemical, Pharmaceutical and Agricultural Sciences (DoCPAS), University of Ferrara, 44121 Ferrara, Italy; 2 Department of Life and Environmental Sciences, Polytechnic University of Marche, 60131 Ancona, Italy; 3 Department of Life Sciences and Biotechnology, University of Ferrara, 44121 Ferrara, Italy; 4 Bavarian Polymer Institute (BPI), Keylab “Electron and Optical Microscopy”, University of Bayreuth, 95440 Bayreuth, Germany; 5 Biotechnology Interuniversity Consortium (C.I.B.), Ferrara Section, University of Ferrara, 44121 Ferrara, Italy

**Keywords:** antioxidant molecules, niosomes, vesicles, hydrogels, topical application, nanoparticle solution X-ray scattering

## Abstract

In the present study, gels based on xanthan gum and poloxamer 407 have been developed and characterized in order to convey natural antioxidant molecules included in niosomes. Specifically, the studies were conducted to evaluate how the vesicular systems affect the release of the active ingredient and which formulation is most suitable for cutaneous application. Niosomes, composed of Span 20 or Tween 20, were produced through the direct hydration method, and therefore, borate buffer or a micellar solution of poloxamer 188 was used as the aqueous phase. The niosomes were firstly characterized in terms of morphology, dimensional and encapsulation stability. Afterwards, gels based on poloxamer 407 or xanthan gum were compared in terms of spreadability and adhesiveness. It was found to have greater spreadability for gels based on poloxamer 407 and 100% adhesiveness for those based on xanthan gum. The in vitro diffusion of drugs studied using Franz cells associated with membranes of mixed cellulose esters showed that the use of a poloxamer micellar hydration phase determined a lower release as well as the use of Span 20. The thickened niosomes ensured controlled diffusion of the antioxidant molecules. Lastly, the in vivo irritation test confirmed the safeness of niosomal gels after cutaneous application.

## 1. Introduction

Antioxidants molecules (AMs) represent our body’s defense system against damage caused by free radicals: they are chemically defined as reducing agents capable of slowing down or preventing the oxidation chain reactions triggered by free radicals in the presence of oxidative stress.

Much of the inputs to the production of radicals occurs as a consequence of the body’s metabolism and respiration, but also psycho-physical stress, environmental factors such as smog and smoke, and UV rays can increase the production of reactive elements inducing a cellular overload (oxidative stress) with altered functionality and consequent cutaneous disorders, e.g., dermatitis, tumors, psoriasis, wrinkles, alopecia, acne and skin aging.

For instance, some research has shown that a high concentration of the hydroxyl radical OH· is the cause of the onset of tumors, following an alteration of the genetic information due to the oxidation of the nitrogenous bases [1]. Instead, the attack of the radicals on the lipid substrates, the main components of the cell membranes, gives rise to allyl bond peroxidation reactions, resulting in cell wall degradation.

Therefore, antioxidants are the best allies in keeping the body healthy and for protecting the skin from the harmful effects of external factors. The mechanism of action of AMs in radical inhibition can be summarized in the following three points: (a) neutralization of reactive species, (b) sequestration of transition metal ions (chelating effect) and (c) enzymes inhibition involved in reactive oxygen species production [2]. From a physiological point of view, the biological system uses endogenous AMs such as catalase, glutathione peroxidase and superoxide dismutase to protect the body. When the endogenous defense systems may not be sufficient, the intake of exogenous AMs by diet (i.e., vitamin C and E), or even by topical or oral administration, becomes necessary.

However, it should be kept in mind that many of these substances are particularly insoluble and unstable at different pH and temperature levels and under environmental stress conditions; therefore, the most effective solution is the topical application through delivery systems such as phospholipid-based (liposomes) and niotenside-based (niosomes) vesicular systems [3] to ensure protection from degradation, thus opening up a vast field in the research and production of suitable cosmetic and pharmaceutical formulations.

In the present study, gallic (GA) and ferulic acid (FA) were considered as example AMs of natural origin due to their activity. Indeed, GA is a potent tyrosinase inhibitor with excellent antioxidant effects, together with antibacterial and anti-inflammatory activity [4,5,6]. Moreover, this natural triphenolic compound is an effective ingredient for the treatment of allergic contact dermatitis [7]. However, due to its poor water solubility, it has rarely been used for dermatologic applications. As for FA, its wide use in skin care products is corroborated by numerous studies demonstrating its protective role in skin structure. Furthermore, FA exhibits low toxicity and optimal absorption through the skin and tends to remain in the bloodstream longer than other phenolic compounds [8]. FA also performs powerful anti-inflammatory, antifibrotic—protective of the vascular endothelium—antiplatelet and antiapoptotic actions [9].

Finally, in order to obtain suitable systems for cutaneous application and delivery of AM embedded in niosomes, this research investigated the development and characterization of gels based on poloxamer 407 and xanthan gum.

## 2. Materials and Methods

### 2.1. Materials

Gallic acid (GA), ferulic acid (FA), cholesterol (CH), Tween 20 (T), Span 20 (S) the copolymers poly(ethylene glycol)-block-poly(propylene glycol)-block-poly(ethylene glycol) poloxamer 188 (P) and poloxamer 407 (pol) were from Sigma-Aldrich (St Louis, MO, USA). All other materials and solvents were from Merck Serono S.p.A. (Rome, Italy).

### 2.2. Niosomes Preparation

Niosomes were prepared by thin-layer hydration with slight modifications (Figure S1) [10]. In detail, non-ionic surfactant and cholesterol in a molar ratio 1:1 were solubilized in a methylene chloride/methanol mixture (1:1, *v*/*v*) and subjected to removal under vacuum of the organic solvent residue using a rotary evaporator (Rotavapor R-200, Büchi Italia, Cornaredo, Italy) obtaining a film on the glass wall that was hydrated using a 2 mg/mL AM aqueous solution, swirled and sonicated, at 60 °C, to give a final concentration of 25 mg/mL in terms of total vesicular components. Borate buffer (B) and Poloxamer 188 (P) solution (2.5% *w*/*w*) were alternatively used as a hydration medium. In Table 1 the detailed composition of each formulation is reported.

### 2.3. Niosomes Characterization

The morphology of niosomes was investigated using a Cryogenic Transmission Electron Microscope (Cryo-TEM). After vitrifying and transferring each sample to a Zeiss EM922Omega transmission electron microscope using a cryoholder (CT3500, Gatan Inc., Pleasanton, CA, USA) [11], specimens were examined with doses of about 1000–2000 e/nm^2^ at 200 kV and images digitally recorded by CCD camera (UltraScan 1000, Gatan Inc., Pleasanton, CA, USA) using GMS 1.4 software (Gatan Inc., Pleasanton, CA, USA) to process the images. During the visualization, the temperature of the sample was maintained below −175 °C.

Small-angle and wide-angle X-ray scattering (SAXS and WAXS) experiments were performed using the Offline Xeuss 3.0 Diamond-Leeds SAXS facility (DL-SAXS) at Diamond Light Source (Harwell, UK). A gallium Excillum metaljet (9.4 keV), coupled with a movable beamstop-less Eiger 2 R 1M placed at the two distances of 100 mm and 2000 mm (for WAXS and SAXS, respectively) was used. The final investigated *Q*-range, (*Q* being the modulus of the scattering vector, defined as 4π sin θ/λ, where 2θ is the scattering angle) extended from 0.003 to 1.6 Å^−1^. The experiment exploited the mail-in service. Niosome samples were prepared in 2 mm polycarbonate capillaries, loaded into a capillary ladder, and analyzed, at 36 °C. Each image had a 2 min collection time, with a 0.4 mm X-ray beam. Two-dimensional (2D) data were corrected for background, detector efficiency and sample transmission, then radially averaged to derive I(*Q*) vs. *Q* curves [12].

Niosomes dimension was measured on aqueous diluted fractions (1:10 by volume) using a Zetasizer Nano S90 (Malvern Instr., Malvern, UK) equipped with a 5 mW helium neon laser with a wavelength output of 633 nm. Plasticware, cleaned with detergent washed and rinsed with milliQ water, was used for measurements made at 25 °C at 90° angle and run time around 180 s. “CONTIN” method [13] was used for interpreting the obtained data.

### 2.4. AMs Content in Niosomes

The encapsulation efficiency (EE) in niosomes, expressed as AM content, was determined after 1 day from vesicles production on a ultracentrifuged 300 µl fraction of the formulation using a Microcon centrifugal filter unit YM-10 membrane (NMWCO 10 kDa, Sigma-Aldrich, St. Louis, MO, USA). Ultracentrifugation was conducted at 8000 rpm for 20 min on a Spectrafuge™ 24D Digital Microcentrifuge (Woodbridge, NJ, USA). After diluting 100 microliters of retentate with methanol (1:10, *v*/*v*) the samples were magnetically stirred for 30 min. Afterwards, the amount of AM was quantified by UV, and the encapsulation parameter was calculated using Equation (1).

EE = AM/T_AM_ × 100 (1)

where AM is the amount of GA or FA determined by UV and T_AM_ is the total AM amount weighed to prepare the formulation. AM quantification was performed by means of a double-ray UV/Vis spectrometer (Lambda19 UV/VIS/NIR Spectrometer, Perkin Elmer, Waltham, MA, USA) operating at 260 nm and 318 nm for GA and FA, respectively, in 1 mL quartz cuvettes. To estimate the AM content in the sample, a previously carried out calibration curve for each compound, was used as reference. The evaluation was performed on day 1, 7, 15 and 30 after production.

### 2.5. In Vitro Diffusion Experiments

AM diffusion was evaluated using Franz cells associated with the mixed cellulose esters membrane. Briefly, dried membranes were immersed in distilled water, at room temperature, for 1 h then mounted on Franz diffusion cells (LGA, Berkeley, CA, USA) exposing a surface area of 0.78 cm^2^ (1 cm diameter orifice). The receptor compartment contained 5 mL of PBS 1X. This solution was stirred at 500 rpm, with the help of a magnetic bar, and thermostated, at 32 ± 1 °C, during all the experiments [14]. Then, 1 mL of each formulation was loaded in the donor compartment in contact with the membrane surface. At predetermined time points between 1 and 8 h, 300 uL of receptor phase was withdrawn and subjected to AM content determination by means of UV/Vis spectrometer. The volume of removed sample was replaced with an equivalent volume of fresh PBS. The AM content was measured in four separate experiments, and the mean values ± standard deviations were estimated. The mean values of AM diffused expressed as µg/cm^2^, were then plotted as a function of time. Diffusion coefficients (J_s_) were calculated from the linear portion of the accumulation curve and expressed as normalized fluxes (J_n_) by dividing by AM concentration in the analyzed form, expressed in mg/mL.

### 2.6. Antioxidant Activity

DPPH radical-scavenging assay was utilized to rapidly evaluate the antioxidant capacity [15]. The DPPH assay estimates the hydrogen donation ability of an antioxidant to convert the stable DPPH free radical to 1,1-diphenyl-2-picrylhydrazyl, which is accompanied by a deep-purple-to-light-yellow colorimetric reaction, which can be evaluated by measuring the percentage reduction of the solution absorbance at 517 nm after the radical reaction with the products under examination. The percentage of radical scavenging capacity was obtained applying Equation (2), as follows:

DPPH radical − scavenging capacity (%) = [1 − (A1 − A2)/A0] × 100(2)

where A0 is the absorbance of the control (without AM), A1 is the absorbance in the presence of the AM, and A2 is the absorbance without DPPH. AM (either solutions or niosomes) at different concentrations (0.750 mL) was added to a methanol solution of DPPH (1.5 mL). The absorbance at 517 nm was obtained by means of a UV–Vis spectrophotometer (Jenway 7305 Spectrophotometer, VWR International Srl, Milan, Italy), as previously described [16]. The IC_50_ values were calculated from the results after applying a previously obtained calibration curve. The obtained values (μg/mL) were the mean ± standard deviation of at least three independent experiments.

The quantitative FRAP assay measures the plasma ferric ion reducing capacity as it is based on the reduction of ferric ions (Fe^3+^) to ferrous ions (Fe^2+^) under acidic conditions in the presence of 2,4,6-tripyridyl-s-triazine (TPTZ) [17]. Indeed, the presence of an AM facilitates the reduction of the Fe^3+^–TPTZ complex to the ferrous form, leading to an intense blue coloration measured at 593 nm (absorption maximum wavelength). Trolox was employed to perform the calibration curves, and therefore, the FRAP antioxidant activity is given as μmol Trolox equivalent/g of compounds.

### 2.7. Niosomal Gel Preparation and Characterization

To obtain thickened formulations, xanthan gum and poloxamer 407 were added to niosomes (Figure S1). To be precise, xg (0.75 %, *w*/*w*) was added to niosomal formulation (xg-niosomes) and handily mixed for 10 min up to complete dispersion [18], while pol-niosomes were prepared by the “cold method” gradually adding an amount of poloxamer 407 (15 % *w*/*w*) to cold water (5–10 °C) magnetically stirred for 3 h and then kept at 5 °C for 12 h up to complete dispersion of poloxamer [19].

The spreading capacity of niosomal gels was evaluated 24 h from gel preparation, at 37 ± 1 °C, to mimic the body temperature conditions [20]. To be precise, 100 mg of formulation was placed on a Petri dish (3 cm diameter) and then subjected to pressure by a glass dish carrying a 10 g mass. The time employed by the gel to completely fill the dish was evaluated.

Spreadability assay was performed three times, and then the mean values ± standard deviations were calculated using Equation (3).

S = m × l/t(3)

where S is the spreadability of the niosomal gel, m is the weight (g) attached on the plate top, l is the diameter (cm) of the plate, and t is the time (s) employed by the gel to completely fill the plate diameter [20].

The in vitro leakage of niosomal gels was determined on rectangular agar slides prepared by adding agar (1.5% *w*/*w*) to a citrate buffer with pH 5.5 and stirred, at 95 °C, until solubilization. Then, the cooled gels agar slides were cut. An amount of 30 mg of gel was placed on the top of an agar slide placed in a Petri plate vertically positioned at 90° on a transparent box wall and maintained, at 37 ± 1 °C. After the gel placement, the time taken by the gel to run along the slide was measured. Gel leakage, expressed as the time traveled by the gel on the plate, was evaluated thrice, and the mean values ± standard deviations were determined.

### 2.8. Patch Test

An in vivo irritation test was performed in order to evaluate the effect of niosomal gels applied in a single dose on intact human skin. The occlusive patch test was conducted at the Cosmetology Center of the University of Ferrara following the basic criteria of the protocols for skin compatibility testing of potentially cutaneous irritant cosmetic ingredients on human volunteers (SCCNFP/0245/99) [21,22,23]. The protocol was approved by the Ethics Committee of the University of Ferrara, Italy (study number: 170583). The test was run on 20 healthy volunteers of both sexes, who gave written consent to the experimentation, excluding subjects affected by dermatitis, with a history of allergic skin reaction or under anti-inflammatory drug therapy (either steroidal or non-steroidal). An aluminum Finn chambers (Bracco, Milan, Italy) carrying 10 mg of xg-niosomes was applied onto the forearm or the back skin, safeguarded with self-sticking tape. The sample formulations were loaded onto the Finn chamber by an insulin syringe and left in contact with the skin surface for 48 h. At 15 min and 24 h after patch remotion and skin cleaning from formulation residues, skin irritative reactions (e.g., erythema and/or edema) were estimated. Specifically, erythematous reactions have been sorted out into three reaction degrees, namely, light, clearly visible, and moderate/serious erythema. Irritative reactions were expressed as percentage with respect to total reactions occurring in volunteers.

## 3. Results and Discussion

### 3.1. Production and Characterization of AMs-Loaded Niosomes

It is well known that the low bioavailability and stability of GA and FA hamper the administration on skin and limit the antioxidant potential of these molecules. In order to overcome these limitations, the encapsulation of GA and FA was investigated by the development of a preformulative study [24,25]. In particular, niosomes have been produced in order to obtain the optimal formulation for GA and FA physico-chemical stability, encapsulation efficiency, controlled release, antioxidant effect and administration on skin. Indeed, in niosomes, the accommodation of amphiphilic drugs can occur between the bilayer and the aqueous core of the vesicles. Niosomes were produced following the thin-layer hydration method with the composition reported in Table 1.

**Figure 1:**
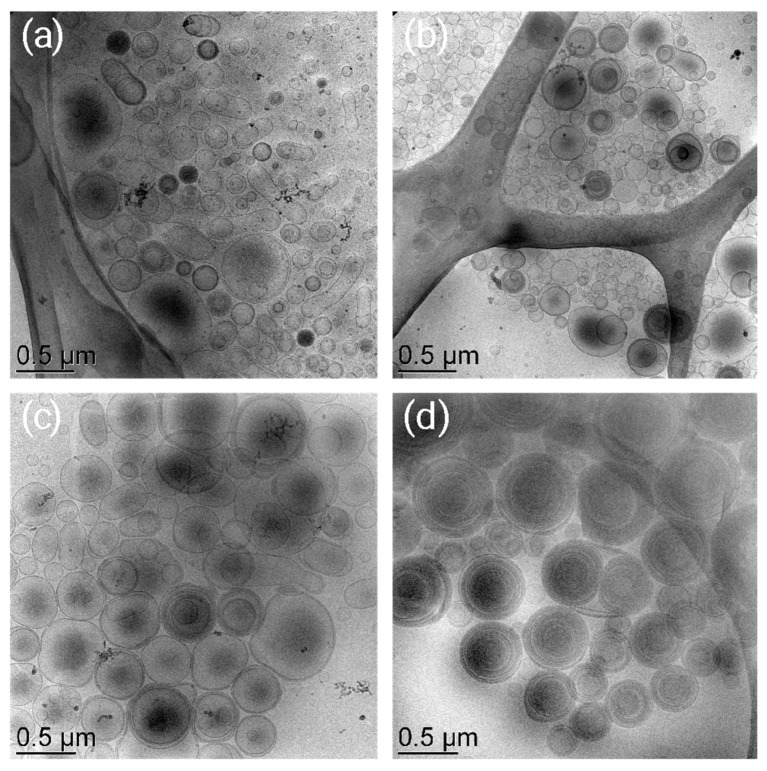
Cryo-TEM images of NSB (**a**), NSP (**b**), NTB (**c**) and NTP (**d**).

Then, the morphology and the inner structure of the produced niosomes were investigated and compared. In particular, the morphological characterization of the different formulations was addressed by Cryo-TEM visualization (Figure 1). Since the presence of the AMs did not alter the aspect of the vesicles, plain niosomes have been considered.

Vesicular systems with different peculiarities ascribable to the components used in the formulation have been obtained. Indeed, uni- or multilamellar non-ionic surfactant vesicles self-assembled in aqueous medium have been obtained [3].

Notably, both S (Figure 1a,b) and T (Figure 1c,d) gave rise to the formation of vesicles, although their lamellarity was affected by the combination between the type of surfactant and the hydration medium. For instance, when B is used, the coexistence of unilamellar, multilamellar and multivesicular systems is evidenced (Figure 1a,c). A similar morphological aspect was obtained for NSP (Figure 1b), while in the case of NTP, multilamellar vesicles were obtained (Figure 1d). This behavior could be ascribed to the interaction between the triblock copolymer p188 (P) and T, and it could be related to the ability of poloxamer micelles to stabilize the vesicular system by creating a “matryoshka” system, as reported in the literature [26].

In order to elucidate the internal structures of the different niosomal formulations, SAXS analysis was performed.

**Figure 2:**
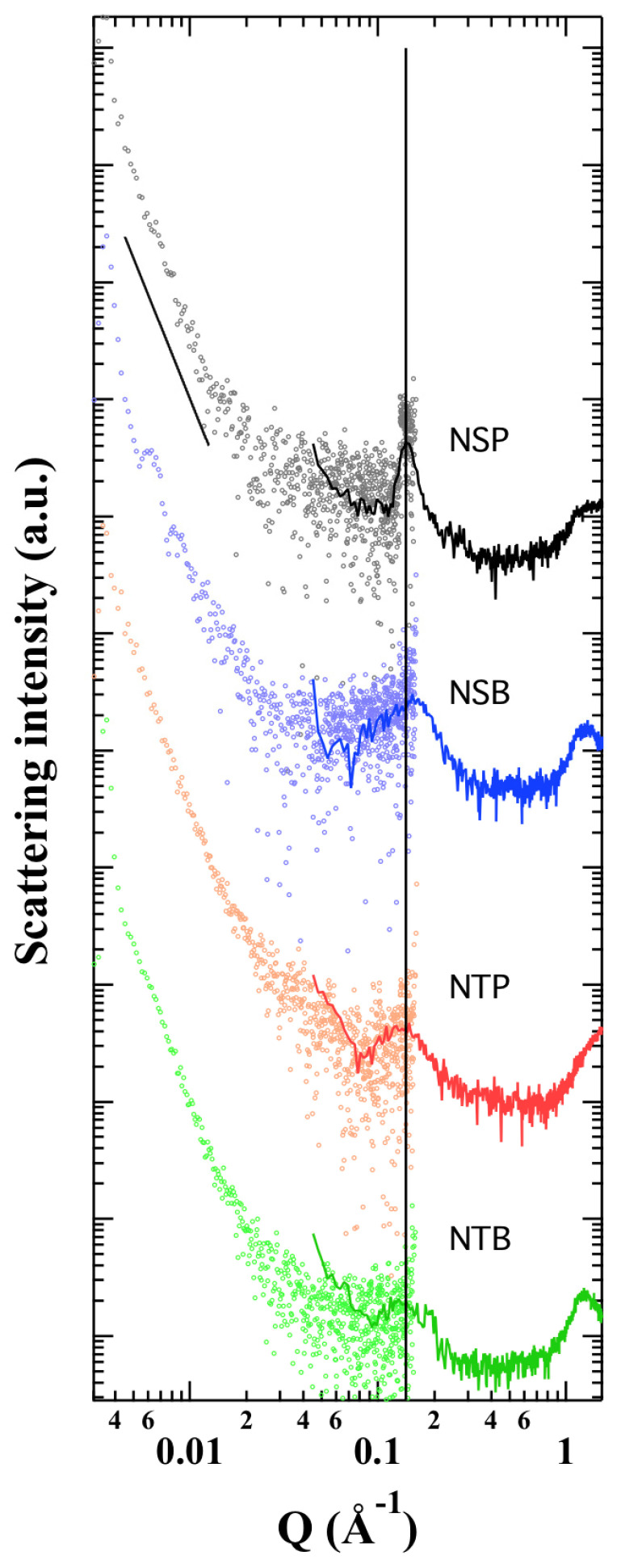
SAXS/WAXS profiles of NSP (black), NSB (blue), NTP (red) and NTB (green).

The results, reported in Figure 2, show similar scattering curves for all the samples, confirming that both S and T determine the formation of similar vesicles. In particular, at a very low angle, the scattering intensities appear to follow a *Q*^−4^-law, suggesting the presence of very large particles (larger than 50–100 nm and probably polydisperse, as detected by Cryo-TEM and PCS), independent from niosomes composition. However, the vesicles show a different degree of lamellarity and a rather disordered nature. Indeed, the Bragg peak observed at about 0.14 Å^−1^ in the scattering profiles confirms the lamellar inner structure of the niosomes, but its width and the absence of the characteristic higher-order peaks suggest that the positional correlation between adjacent bilayers is very small and/or that the degree of lamellarity is very low [27]. Interestingly, both the position and width of the peak appear related to the niosomes composition. As shown in Table 2, the unit cell parameter (which corresponds to the thickness of the vesicles layer plus the thickness of the aqueous layer separating two vesicles layers and which can be calculated from the position of the Bragg peak) depends on the used surfactant, while the degree of lamellarity, which can be indicatively derived by applying the Scherrer equation to the Bragg peak broadening under the assumption that other effects (such as stacking disorder or undulation effects) can be ignored [28], seems controlled by a combination of the type of surfactant and the hydration medium.

Table 2 shows that T probably induces a larger hydration of the vesicular layers (both in the presence of B and of P), so that the total thickness increases by about 10%. On the other side, the presence of P induces an ordering of the lamellar structure, which results in the enhancement of the mean crystallite size, particularly evident in the case of niosomes prepared with S as surfactant.

After production, the AMs-loaded formulations were characterized in terms of size and polydispersity. Afterwards, the dimensional stability was investigated for 30 days, as reported in Table 3.

The size distribution of niosomes after production ranged between 420 and 860 nm; thus, this parameter was strongly influenced by the composition of the vesicles. In particular, the higher dimensional increase was evidenced for niosomes comprising the combination of T and P as a surfactant and hydrating phase. This data agrees with the morphological and SAXS analyses, as well as with the literature [26], supporting the hypothesis that the interaction between the micelles and the bilayers could facilitate the formation of larger vesicles [26]. Indeed, as mentioned above, the resulting dimensions are influenced not only by the nature of T, responsible for the greater hydration of the layers, but also by the increase in lamellarity induced by P.

Moreover, no significant differences related to the alternative encapsulation of each AM have been evidenced.

Concerning dispersity indexes, the presence of heterogeneous populations has been denoted, in particular, one month after production, since PdI values higher than 0.2 indicate a wide size distribution [29].

### 3.2. Encapsulation Efficiency of Ams

**Figure 3:**
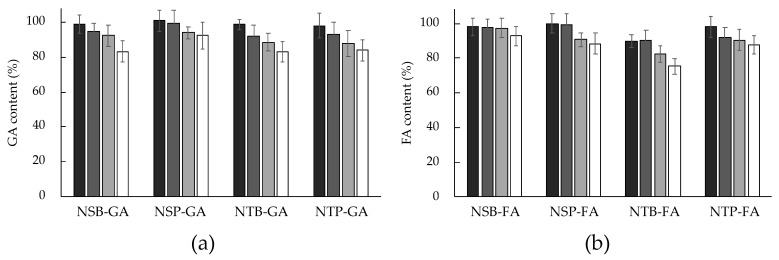
Percentage of loaded AMs in niosomes ((**a**), GA; (**b**), FA) at day 1 (black), 7 (dark grey), 15 (light grey) and 30 (white) after production. Data are the average of four independent experiments ± s.d.

The percentage of Ams content in the different vesicular systems was evaluated over time, and the chemical stability of the loaded drug was monitored up to 30 days after production. Figure 3 shows the drug content in niosomes, expressed as a percentage of the total amount used in the formulations.

The results reveal that niosomes allowed a quantitative encapsulation of AMs after production, reaching percentages between 90% and 100% independently of the type of formulation.

Specifically, in the case of GA, the composition of the formulations did not affect the encapsulation efficiency of the drug, which was maintained stable, reaching content values around 83% one month after production. The stability profile of GA content was similar for all the formulations. Concerning FA, NSB, NSP and NTP retained more than 85% of the drug after one month, while for NTB, the amount of drug decreased, reaching 75% of encapsulation. This behavior suggests that the presence of S, especially in combination with B as a hydration medium, ensured better stability to the system, as observed also in terms of size. Notably, the role of S as a destabilizing agent, when in aqueous solution, facilitates an increase in the entrapment efficiency of drugs, possibly explained by its HLB value being 8.6 [30].

### 3.3. In Vitro Diffusion Kinetics

**Figure 4:**
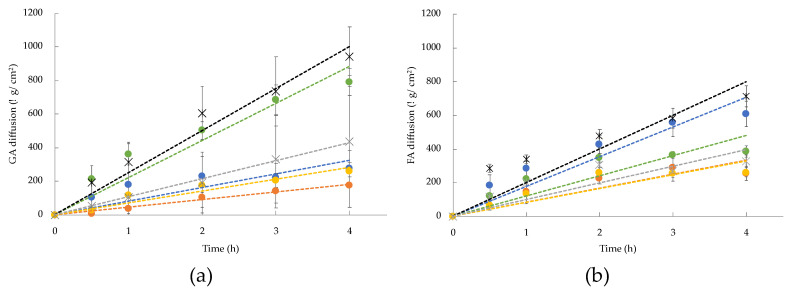
In vitro diffusion profiles of GA (**a**) and FA (**b**) from B (black cross), P (grey cross), NSB (blue), NSP (orange), NTB (green) and NTP (yellow). Data are the mean of four unrelated experiments ± s.d.

The effect of the niosomes composition on AM diffusion has also been investigated by means of Franz-cell experiments. To be precise, to in vitro mimic the physiological conditions of AM diffusion through the skin, mixed cellulose esters membrane [31] and phosphate-buffered saline (pH 7.4) as receiving phase were employed. The amount of AM in the receiving phase was quantified by UV spectrometry, and the diffusion of the drug through the membrane was plotted against time, expressed as μg/cm^2^ and hours, respectively. In Figure 4, the diffusion of GA (panel A) and FA (panel B) from niosomes and from two different reference solutions, namely, B and P, are displayed. The linear profile within 4 h was considered.

As expected, from the comparison between each reference solution (crosses) and the corresponding niosomal formulation (dots) in terms of the aqueous phase, a controlled diffusion of AM from the vesicles has been evidenced. This behavior was more pronounced for B as compared to P. However, also for P, the solution ensured a controlled diffusion, ascribable to the chemical nature of the co-polymer.

Furthermore, considering the hydration medium, it should be noticed that NSB and NTB displayed a faster drug diffusion profile with respect to those from NSP and NTP for both AMs. Indeed, the use of a micellar solution in the formulation conferred a “matryoshka” configuration to the system [26], responsible for the controlled release of the drug that can be loaded into the micelles and simultaneously into the internal core of vesicles. It is supposed that in this case, poloxamer 188, employed as a hydrating phase, retained the drug encapsulated in the micelles, allowing a slower diffusion of the drug through the membrane.

### 3.4. Antioxidant Activity

**Figure 5:**
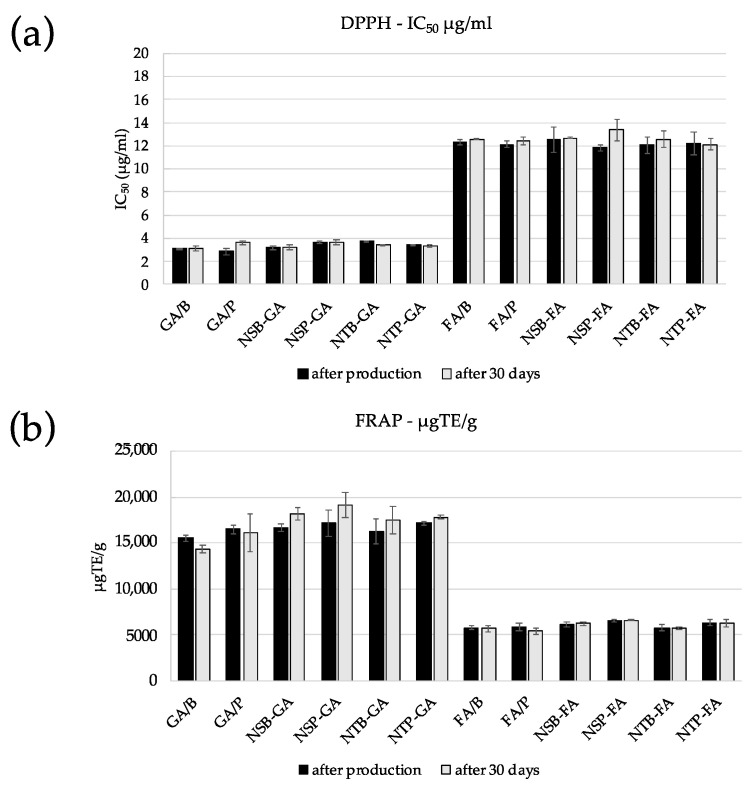
Antioxidant activity of GA and FA containing solutions and formulations as determined by DPPH (**a**) and FRAP (**b**) assays, 1 (black) and 30 (light gray) days after production.

The use of GA and FA as antioxidants, in particular as radical-scavenging agents, is well demonstrated in the cosmetic and pharmaceutical fields [8,32,33,34]. In this study, DPPH assay was performed to compare the radical scavenging activity of the encapsulated AM by considering the IC_50_ values and FRAP assay to investigate their total antioxidant potential. Stability, intended as maintenance of the antioxidant effect, was monitored up to 30 days after production, and the results are showed in Figure 5.

Comparing the different niosomal formulations with the corresponding AM solutions, the data obtained showed that the encapsulation strategy was able to maintain the antioxidant potential of the molecules, confirming the suitability of niosomes as GA and FA delivery systems. Regarding the DPPH assay, as can be seen in Figure 5a, the activity profile is higher for GA (IC_50_ values in the range 2.87–3.77 μg/mL) than for FA (IC_50_ values in the range 11.83–12.51 μg/mL).

NTB-GA displayed higher antioxidant activity than GA/B solution (IC_50_ values were significantly different; *p* < 0.0001). Additionally, NSP-GA and NTP-GA showed higher and statistically significant IC_50_ values than GA/P (*p* < 0.012 and *p* < 0.025, respectively). In contrast, all FA-containing formulations possessed comparable antioxidant capacity to FA/B (non-statistically significant differences).

In general, the IC_50_ values monitored one month after production were maintained during the observation period without statistically significant changes, with the exception of NTB-GA, which showed a statistically significant decrease in activity (*p* < 0.0001).

Regarding the FRAP assay, the results corroborated what was shown by DPPH, indicating a greater antioxidant effect for GA (μmol TE/g ranging from 15,530.70–17,142.54) than FA (μmol TE/g ranging from 5714.88–6468.45) (Figure 5b). In particular, NSB-GA and NSP-FA showed higher antioxidant capacity than the reference GA/B and FA/B solutions (µgTE/g values were significantly different: *p* < 0.023 and *p* < 0.046, respectively). The 30-day stability of FRAP activity was maintained, except for GA/B, NSB-GA and NTP-GA, which showed a statistically significant decrease (*p* < 0.019, *p* < 0.031, and *p* < 0.031, respectively).

### 3.5. Niosomal Gel Production and Technological Behavior

Being niosomes dispersed in liquid form, the administration onto the skin is difficult and onsite permanence limited. Therefore, to obtain formulations with a certain grade of viscosity suitable for topical application, two different gelling agents, namely, xanthan gum and poloxamer 407 have been selected and added to niosome formulations. Indeed, these two biocompatible polymers are able to confer different adhesive properties to niosomes. Xanthan gum, an anionic polysaccharide obtained by bacterial fermentation [35], is a good thickening agent, while on the other hand, poloxamer 407 possesses peculiar thermo-reversible properties passing from a sol state at a low temperature to gel consistency at body temperature [31]. Spreadability and leakage were investigated in vitro to verify the suitability of the thickened formulations for topical administration. Indeed, these parameters are involved in many properties of the final formulation, such as gel extrusion from the package, skin coverage, patient compliance and drug therapeutic efficacy [18]. It has been demonstrated that presence of encapsulated drugs does not affect the technological performance of the thickened systems [18]; therefore, experiments were performed on empty formulations. The obtained results are reported in Table 4.

As evidenced, the addition of xanthan gum led to the formation of a stiff gel formulation. In fact, concerning spreadability, the lowest values have been obtained on xg-niosomes, and no running across the agar plate has been observed in leakage tests. This aspect could ensure higher retention on the application site. Conversely, pol-niosomes showed higher values in terms of spreadability and leakage behavior.

In general, considering the hydration medium, niosomes composed of P resulted in thicker formulations, independently of the gelling agent used. This result could be related to the above-described role of the P micellar system in creating a rigid and complex structure.

Concerning the pH characteristics of niosomal gels, as summarized in Table 5, it emerges that the use of pol as a hydration medium gives rise to the formation of a pH around 5, but this is compatible anyway with skin characteristics. Taking into consideration the pKa values of both GA (4.41) and FA (4.58) we can hypothesize that the ratio at pH 5 between GA and gallate or FA and ferulate may be shifted towards the undissociated form. Indeed, from a micro and macroscopical point of view, no aggregation, precipitation, separation or changes in the aspect of the gelled formulation are evident. Therefore, we assume that the presence of these levels of dissociated form of AM is not able to induce changes in the solubility of the gelling polymers (i.e., poloxamer 407 and xanthan gum) and on the characteristics of the final topical formulations. Furthermore, many studies have shown no difference in antioxidant activity between ionized and non-ionized forms of the AMs [36,37,38,39,40].

### 3.6. AMs Diffusion from Niosomal Gels

**Figure 6:**
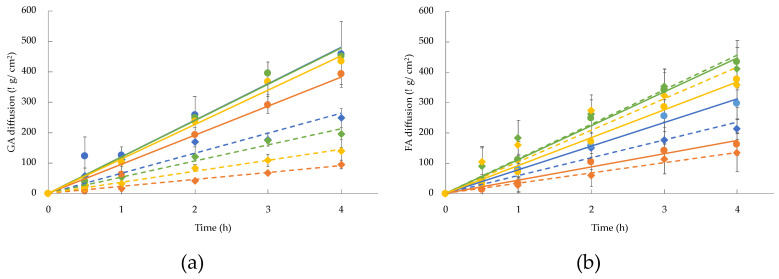
In vitro diffusion profiles of GA (**a**) and FA (**b**) from NSB (blue), NSP (orange), NTB (green) and NTP (yellow), when thickened with xg (dashed line, diamond) or pol (continue line, dot). Data represent the mean of four independent experiments ± s.d.

The AM diffusion from niosomal gels, obtained after addition of xg or pol, has been investigated and compared. In Figure 6, diffusion profiles of GA (panel A) and FA (panel B) from niosomal gels are shown considering the linear portion within 4 h.

As depicted, the obtained diffusion profiles showed different behavior for GA and FA. In the case of GA, the passage of the drug through the membrane is mainly influenced by the thickening agent. In fact, the addition of xg provides a higher control on drug diffusion with respect to pol.

Concerning FA, the different behavior seems to be related to the nature of the surfactant employed for the formulation. In the case of NSB and NSP, xg induced a lowest FA passage, while for NTB and NTP no significant differences have been detected with the use of xg or pol. Furthermore, the diffusion kinetics of AMs were expressed as fluxes (Table 6), extrapolated from the slopes of diffusion profiles (J_s_) and normalized in function of the experimental AM concentration in each formulation (J_n_).

Concerning GA, the pol-made gel led to a faster diffusion of the drug. In fact, the possible solubilization of GA in pol, thanks to the ability of the polymer in creating a micellar network, could be responsible for its increased passage through the membrane.

Considering the J_n_ values of FA diffusion, it was found that when T is used in the composition, the addition of a thickening agent did not affect a controlled passage of the drug with respect to formulations and data are superimposable. This behavior could be ascribed to the interaction between T and FA, and thus, it can be proposed that the diffusion is mainly governed by the vesicular system and less by the hydrophilic nature of the polymers, resulting in less structured gels.

In all cases, P as a hydration medium of niosomes plays a crucial role in GA diffusion, corroborating the influence of the “matryoshka” configuration on drug diffusion.

### 3.7. Patch Test

**Figure 7:**
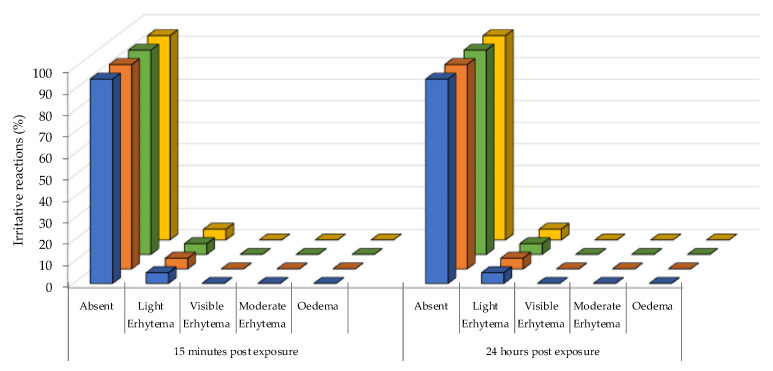
In vivo irritative reactions of xg-NSB (blue), xg-NSP (orange), xg-NTB (green) and xg-NTP (yellow) on 20 healthy volunteers, acquired by patch test.

Being the topical administration the final target of these formulations, a patch test was performed in order to evaluate the safeness of the produced niosomal gels. Taking into account the results of spreadability and leakage, xg-niosomes have been selected for the patch test. To be precise, the potential irritation caused by applying xg-NSB, xg-NSP, xg-NTB and xg-NTP on skin have been evaluated on 20 healthy volunteers and the results expressed as a percentage of irritative reactions (Figure 7).

It was found that the formulations exhibited the same results in terms of safeness; thus, all niosomal gels applied for 48 h under occlusive conditions can be classified as non-irritant. In fact, in 95% of cases, no irritative reactions have been detected, while 5% of cases led to negligible reactions, confirming the suitability of these systems for topical application.

## 4. Conclusions

The present investigation enabled the design of niosomal gels suitable for AMs loading and subsequent topical delivery. Specifically, this study highlighted the influence of a hydration medium (B or P) and a thickening agent (xg or pol) on the diffusion of AMs.

Strikingly, the results of the Franz-cell experiments and patch tests demonstrated that niosomal gel can deliver AMs in different ways depending on the interaction between the loaded drug, the non-ionic surfactant and the thickener agent. Indeed, in the case of FA, the presence of T facilitated an in vitro diffusion mainly governed by the vesicular system, while in the case of GA, an important role is due to the hydrophilic nature of the thickeners, corroborating the influence of the “matryoshka” configuration on drug diffusion. These data are in agreement with the cryo-TEM and SAXS analyses, showing the coexistence of unilamellar, multilamellar and multivesicular systems after hydration with B, or sole multilamellar vesicles, in the case of P hydration. However, an important contribution of the surfactant constituting the niosomes has been detected by means of SAXS analysis. Indeed, T seems to give rise to great hydration of the vesicle’s layers both in the presence of B and of P, while in the case of niosomes prepared with S as the surfactant, the presence of P induces an ordering of the lamellar structure resulting in the enhancement of the mean crystallite size.

Moreover, no irritative reactions were detected during patch tests in 95% of cases, confirming the suitability of these systems for topical application.

## Figures and Tables

**Table 1:** Composition of the produced niosomes.

Acronym	Composition					
	Molar Ratio			Aqueous Phase	Organic Phase (mg/mL)	AM (mg/mL)
	Cholesterol	Span 20	Tween 20			
GA/B; FA/B	-	-	-	Borate Buffer (B)	-	2
GA/P; FA/P	-	-	-	Poloxamer 188 * (P)	-	2
NSB-GA; NSB-FA	1	1	-	Borate Buffer (B)	25	2
NSP-GA; NSP-FA	1	1	-	Poloxamer 188 * (P)	25	2
NTB-GA; NTB-FA	1	-	1	Borate Buffer (B)	25	2
NTP-GA; NTP-FA	1	-	1	Poloxamer 188 * (P)	25	2

* 2.5% *w*/*w*.

**Table 2:** Structural parameters for the uni-/multilamellar niosomes, as determined by SAXS/WAXS.

Niosome Acronym	Peak Position (2θ)	Peak Width (2θ)	Unit Cell *d* (nm)	Mean Crystallite Size *L* (nm)	Approximate Number of Interacting Bilayer *L*/*d*
**NSP**	1.299	0.129	4.41	41.7	9
**NSB**	1.290	0.554	4.44	9.7	2
**NTP**	1.180	0.388	4.86	13.9	3
**NTB**	1.191	0.681	4.81	7.9	2
***errors***			*± 0.10*	*± 20%*	

**Table 3:** Dimensional behavior overtime of AMs-loaded niosomes, as determined by PCS.

Time (d)	NSB-GA	NSP-GA	NTB-GA	NTP-GA	NSB-FA	NSP-FA	NTB-FA	NTP-FA
	Z-Ave (nm)	Z-Ave (nm)	Z-Ave (nm)	Z-Ave (nm)	Z-Ave (nm)	Z-Ave (nm)	Z-Ave (nm)	Z-Ave (nm)
	*PdI*	*PdI*	*PdI*	*PdI*	*PdI*	*PdI*	*PdI*	*PdI*
1	549 ± 49	456 ± 28	436 ± 39	610 ± 16	419 ± 61	594 ± 4	495 ± 41	862 ± 53
	*0.13 ± 0.07*	*0.19 ± 0.07*	*0.15 ± 0.03*	*0.25 ± 0.01*	*0.27 ± 0.10*	*0.36 ± 0.02*	*0.27 ± 0.03*	*0.39 ± 0.05*
7	614 ± 35	573 ± 35	451 ± 32	686 ± 31	373 ± 21	499 ± 39	473 ± 42	895 ± 27
	*0.11 ± 0.02*	*0.30 ± 0.03*	*0.26 ± 0.10*	*0.40 ± 0.05*	*0.31 ± 0.07*	*0.27 ± 0.03*	*0.28 ± 0.01*	*0.35 ± 0.03*
15	613 ± 50	604 ± 44	501 ± 14	825 ± 26	377 ± 25	456 ± 38	498 ± 34	932 ± 31
	*0.17 ± 0.03*	*0.24 ± 0.04*	*0.41 ± 0.03*	*0.28 ± 0.09*	*0.22 ± 0.09*	*0.23 ± 0.08*	*0.30 ± 0.01*	*0.33 ± 0.08*
30	601 ± 46	779 ± 37	546 ± 28	1021 ± 48	479 ± 29	422 ± 26	526 ± 23	1082 ± 98
	*0.21 ± 0.01*	*0.26 ± 0.02*	*0.31 ± 0.02*	*0.29 ± 0.07*	*0.25 ± 0.03*	*0.24 ± 0.01*	*0.29 ± 0.02*	*0.32 ± 0.15*

**Table 4:** Spreadability values and leakage behavior of niosomal gel obtained by the addition of xanthan gum (xg) and poloxamer 407 (pol) to the formulations.

Acronym	Spreadability (g·cm/s)	Leakage (s)
**xg-NSB**	3.10 ± 0.25	n.d.
**xg-NSP**	1.52 ± 0.09	n.d.
**xg-NTB**	3.02 ± 0.19	n.d.
**xg-NTP**	1.03 ± 0.07	n.d.
**pol-NSB**	8.93 ± 0.52	8.49 ± 0.38
**pol-NSP**	4.18 ± 0.29	7.16 ± 0.42
**pol-NTB**	9.77 ± 0.64	8.23 ± 0.48
**pol-NTP**	4.08 ± 0.22	7.36 ± 0.32

n.d.: not detectable.

**Table 5:** pH values of niosomal formulations before and after gelification obtained by the addition of xanthan gum (xg) or poloxamer 407 (pol).

Acronym	pH		
	Niosomes ^1^	xg-Niosomes ^2^	pol-Niosomes ^3^
**NSB-GA**	4.7 ± 0.5	5.2 ± 0.3	5.4 ± 0.1
**NSP-GA**	4.9 ± 0.1	4.8 ± 0.1	4.9 ± 0.2
**NTB-GA**	5.0 ± 0.1	4.9 ± 0.1	5.9 ± 0.1
**NTP-GA**	4.7 ± 0.1	4.9 ± 0.2	5.4 ± 0.2
**NSB-FA**	6.0 ± 0.3	6.3 ± 0.3	6.5 ± 0.1
**NSP-FA**	5.2 ± 0.3	5.1 ± 0.2	5.6 ± 0.1
**NTB-FA**	6.0 ± 0.1	5.9 ± 0.2	6.0 ± 0.2
**NTP-FA**	5.5 ± 0.2	5.4 ± 0.2	5.3 ± 0.2

^1^ niosomes formulation; ^2^ niosomes thickened with xg; ^3^ niosomes thickened with pol.

**Table 6:** Diffusion coefficients of GA and FA.

Acronym	GA		FA	
	J_s_ (μg/cm^2^·h)	J_n_ (cm^2^·h)	J_s_ (μg/cm^2^·h)	J_n_ (cm^2^·h)
**NSB**	81.0	40.5	176.7	88.3
**xg-NSB**	66.0	33.0	58.9	29.4
**pol-NSB**	120.0	60.0	78.2	39.1
**NSP**	45.3	22.7	83.3	41.6
**xg-NSP**	22.6	11.3	34.1	17.0
**pol-NSP**	95.7	47.8	43.8	21.9
**NTB**	220.7	110.4	119.5	59.8
**xg-NTB**	53.1	26.6	114.4	57.2
**pol-NTB**	119.2	59.6	112.1	56.1
**NTP**	70.3	35.1	82.4	41.2
**xg-NTP**	36.2	18.1	104.5	52.3
**pol-NTP**	112.9	56.5	92.3	46.2

## Data Availability

Not applicable.

## Supplementary Materials

The following supporting information can be downloaded at: https://www.mdpi.com/article/10.3390/gels9020107/s1, Figure S1: Graphical diagram of the production steps of niosomes and niosomal gels.
