# Supplementary material for: Niosomes for Topical Application of Antioxidant Molecules: Design and In Vitro Behavior
Source: Gels. 2023 Jan 26;9(2):107. doi: 10.3390/gels9020107 (PMC9956392; doi:10.3390/gels9020107)
Supplement: Supplementary file 1 [file gels-09-00107-s001.zip › gels-2164505-supplementary.pdf]

gels

*Supplementary Material*

# Niosomes for Topical Application of Antioxidant Molecules: Design and In Vitro Behavior

**Maddalena Sguizzato [1], Alessia Pepe [2], Anna Baldisserotto [3], Riccardo Barbari [3], Leda Montesi [3], Markus Drechsler [4], Paolo Mariani [2] and Rita Cortesi [1,5,*]**

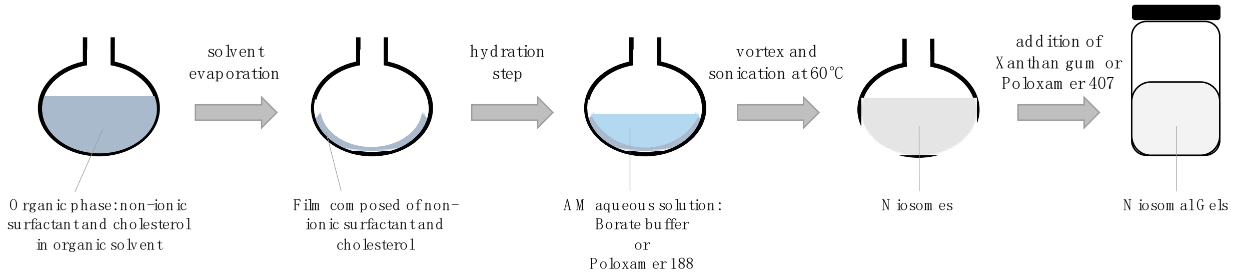


**Figure S1.** Graphical diagram of the production steps of niosomes and niosomal gels.
